# Lobetyolin reshapes gut microbiota and bile acid metabolism to improve androgen-driven PCOS phenotypes in mice

**DOI:** 10.3389/fmicb.2026.1810261

**Published:** 2026-05-01

**Authors:** Lijuan Li, Pingping Lin, Siyuan Liu, Yuning Zhang, Siqi Wang, Jingtao Liang, Ruyu Wang, Guofeng Duan, Aixiang Chen

**Affiliations:** 1 Shanxi Provincial Department-Municipal Key Laboratory Cultivation Base for Quality Enhancement and Utilization of Shangdang Chinese Medicinal Materials, Changzhi Medical College, Changzhi, Shanxi, China; 2 School of Nursing, Changzhi Medical College, Changzhi, Shanxi, China

**Keywords:** bile acid metabolism, gut microbiota, lobetyolin, polycystic ovary syndrome (PCOS), VEGFA/VEGFAR2

## Abstract

**Aim:**

To clarify the molecular mechanisms through which lobetyolin (LT) modifies the gut microbiota-bile acid axis to multitarget the regulation of hyperandrogenism, insulin resistance, chronic low-grade inflammation, and endometrial dysfunction, consequently breaking the vicious cycle and improving the pathological phenotype of polycystic ovary syndrome (PCOS), thereby establishing a theoretical basis and experimental validation for the advancement of LT as a systemic therapeutic agent for PCOS.

**Methods:**

A DHEA-induced PCOS murine model was administered LT (i.p. for 28 days). We monitored hormone levels in the serum, glucose tolerance, and estrus cycle. Ovarian steroidogenic enzyme expression (CYP11A1, CYP17A1, and CYP19A1) was assessed by qPCR, while inflammatory markers (IL-6, TNF-α, TLR4, and NF-κB) were quantified by ELISA.16S rRNA sequencing was performed for gut microbiota and focused bile acid metabolomics. Uterine molecular markers were evaluated through vascular endothelial growth factor A (VEGFA)/vascular endothelial growth factor receptor (VEGFR2), tissue inhibitor of metalloproteinases 1(TIMP1/2), and matrix metalloproteinase 2/9 (MMP2/9).

**Main results:**

LT restored estrous cyclicity, lowered the LH/FSH ratio and serum testosterone levels, and improved insulin sensitivity. Androgen Network Modulation: LT decreased CYP11A1/CYP17A1 and elevated CYP19A1, showing “upstream inhibition-downstream activation.” LT also boosted α-diversity, adjusted the F/B ratio, enriched *Dubosiella*/*Muribaculum*, and inhibited *Lachnospiraceae and Alistipes*. Higher HCA and TCDCA levels are associated with improved metabolism. LT alleviated pathological vascular remodeling in the uterus by downregulating VEGFA/VEGFR2 and MMP2/9, and by overexpressing TIMP1/2.

**Conclusion:**

LT modulates the microbiota-bile acid axis to coordinately ameliorate hyperandrogenism, insulin resistance, chronic inflammation, and uterine dysfunction. This intervention blocks the pathological cascade of PCOS. The findings provide a subsequent mechanistic studies on PCOS prevention.

## 1 Introduction

Approximately 8% and 13% of women of reproductive age have polycystic ovarian syndrome (PCOS), the most common heritable endocrine metabolic disorder. The condition is characterized by hyperandrogenism, oligo-ovulation, and polycystic ovaries, with 70% of cases going undetected (Dou et al., 2024; Salehi et al., 2024). Insulin resistance, lipotoxicity (LPS), low-grade inflammation, and oxidative stress are also associated with the most common causes of anovulatory infertility. These factors increase the risk of cardiovascular disease, endometrial cancer, and type 2 diabetes (Rizk et al., 2024; Liu Z. Q. et al., 2024). Although the origin is uncertain, endocrine metabolic illnesses may be linked to follicular developmental arrest due to an imbalance in the “angiogenesis-extracellular matrix (ECM) remodeling” axis of the ovarian local microenvironment (Duan et al., 2021; Olaizola et al., 2022). A crucial signaling module consisting of VEGFA and VEGFR controls the transition from preantral to antral follicles and ovarian vascular permeability (Li W. et al., 2021). According to clinical and animal studies, individuals with PCOS have higher serum and ovarian tissue VEGFA levels, which are favorably correlated with both testosterone concentration and the number of antral follicles (Dambala et al., 2017). (Shalaby et al. 2024) discovered that downregulating the VEGFA/VEGFR2 signaling pathway decreased ovarian hyperemia and cyst formation in a letrozole-induced PCOS rat model, suggesting its therapeutic potential. Follicle wall rupture during extracellular matrix (ECM) remodeling is determined by the dynamic balance between matrix metalloproteinases (MMPs) and their endogenous tissue inhibitors (TIMPs) (Ranjbaran et al., 2016). Higher MMP2 and MMP9 activity in women with PCOS was first observed by (Lewandowski et al. 2006). This axis may be involved in the last follicular maturation arrest, as Gomes et al. discovered that an imbalance in the MMP2/9 to TIMP1/2 ratio could predict the susceptibility of patients with PCOS to ovulation-inducing drugs (Gomes et al., 2011). The substantial correlation between MMP2/9 concentrations and waist circumference, HOMA-IR, and free androgen index has been confirmed in other independent cohorts (Han et al., 2023; Adhikari et al., 2024). Stromal fibrosis is correlated with the local upregulation of these factors in the ovaries (Gu et al., 2024). TIMP changes in PCOS are controversial; some studies have found downregulation of TIMP1/2 in follicular fluid, while other studies have found compensatory increases (Gomes et al., 2011; Rahat et al., 2016; Baka et al., 2010). These discrepancies could be explained by the detection methods, phenotypic heterogeneity, and sample source.

The gene repertoire of the gut microbiota is at least 150 times larger than that of the human host, making it a “virtual endocrine organ.” It communicates with the host in both directions through processes such as energy collection, immunological education, and signaling via secondary metabolites (Escorcia Mora et al., 2025). Recent cross-sectional and longitudinal cohort studies conducted in the last 5 years have suggested that people with PCOS have “low-grade dysbiosis” (Li Y-.X. et al., 2025), which is characterized by a general decrease in α-diversity and a shift in β-diversity toward a proinflammatory phenotype (Li J. et al., 2025). Certain studies have found a lower proportion of *Firmicutes* and a higher proportion of *Bacteroidetes*, resulting in a lower F/B ratio (Rodriguez Paris et al., 2022), whereas other studies have found an elevated or no discernible difference in the F/B ratio (Zhou et al., 2023). The relative abundance of potentially harmful genera, such as *Fusobacterium, Porphyromonas*, and *Prevotella*, is markedly elevated in patients with PCOS, whereas *Lactobacillus* and *Bifidobacterium*, which are involved in butyrate production and barrier maintenance, exhibit synchronous depletion (Salehi et al., 2024; Li C. et al., 2025; Parua et al., 2025; He and Li, 2020). In addition to having a positive correlation with androstenedione levels and the homeostasis model assessment of insulin resistance (HOMA-IR), this imbalance between “proinflammatory” and “anti-inflammatory” taxa is accompanied by elevated levels of circulating LPS, indicating that changes in microbial structure may be a major factor in the metabolic reproductive phenotype of women with PCOS.

Lobetyolin, a polyacetylene glycoside extracted from the roots of *Codonopsis pilosula*, is a crucial quality indicator of this herb (Bailly, 2021; Wang J. et al., 2023). Prior research has shown that this molecule possesses bioactivity across multiple signaling pathways, including anticancer, antioxidant, anti-inflammatory, and immunomodulatory properties (Wang S. S. et al., 2023; Xie et al., 2023). In an OGD/R-induced BV2 cell injury model, lobetyolin significantly inhibited the secretion of proinflammatory factors, such as TNF-α and IL-6, downregulated the expression of inducible nitric oxide synthase (iNOS), and facilitated the M1-to-M2 phenotypic switch, thereby mitigating neuroinflammatory damage (Wang J. et al., 2023). The crude extract of *Codonopsis pilosula* inhibits α-glucosidase, with lobetyolin being the principal active constituent (Jia et al., 2022). Recent studies have demonstrated that lobetyolin diminishes low-grade intestinal inflammation and rectifies lipid metabolic disorders in hyperlipidemic mice by altering gut microbiota composition (Duan et al., 2025). Nonetheless, in the clinical setting of PCOS, marked by metabolic and reproductive dysfunction, it is uncertain whether lobetyolin affects disease progression through the “microbiota-bile acid-angiogenesis” axis. Clarifying this method would enhance our understanding of the multitarget characteristics of monomeric constituents in traditional Chinese medicine and provide a distinctive chemical probe for targeted therapy of PCOS.

## 2 Materials and methods

### 2.1 Major instruments and equipment

The following equipment was utilized in the study: a high-speed refrigerated centrifuge (Sigma, Germany), ultra-low temperature freezer (Thermo Fisher Scientific, Shanghai, China), paraffin microtome (Leica Biosystems Nussloch GmbH, Germany), thermal cycler (Analytik Jena AG, Germany), automated nucleic acid purification system (Qiagen, Germany), microscopic image analysis system (Olympus Corporation, Japan), and blood glucose meter (Shanghai Yuyan Scientific Instruments Co., Ltd., China).

### 2.2 Major chemical agents and reagents

Lobetyolin (purity ≥ 98%, Chengdu Gelip Biological Technology Co., Ltd., China); dehydroepiandrosterone (DHEA; Beijing Zhongke Hengyi Technology Co., Ltd., Beijing, China); sesame oil (Sigma-Aldrich); and FINS, LH, TSH, T, and PRL ELISA kits (Sangon Biotech Co., Ltd., China); GSH, SOD, and MDA Kits (Nanjing Jiancheng Bioengineering Institute, China); Hematoxylin-Eosin (H&E) Staining Kit (Acmec Biochemical, Shanghai, China); IL-6, LPS, IL-4, IL-10 ELISA Kits (Xiamen Luncangshuo Biotechnology Co., Ltd., China); SYBR Green PCR Master Mix (Takara Bio, Japan); TrIzon One-Step gDNA Removal Kit (Servicebio Technology Co., Ltd., Wuhan, China), and qPCR primers were procured from Servicebio Technology Co., Ltd. (Wuhan, China), and 4% Tissue Cell Fixative Solution (Beijing Dingguo Changsheng Biotechnology Co., Ltd., China). All chemicals and solvents used were of the highest commercially available grade.

### 2.3 Animals experiment

Beijing-based (SPEPharm Biotechnology Co. Ltd., Beijing, China) we obtained 4-week-old female Kunming mice. After 1 week of untreated adaptive feeding. All mice were enrolled in the study in numerical order and randomly allocated to experimental groups by lottery, with the group allocation performed by a third-party investigator to ensure that the experimenters remained blinded to group assignments. Model induction for DHEA: Mice in the Model group were subjected to daily subcutaneous injections of DHEA (60 mg/kg body weight), dissolved in sesame oil, for 4 weeks to create a model similar to polycystic ovary syndrome. LT injections were administered at doses of 10 mg/kg body weight to the LT1 group and 50 mg/kg body weight to the LT2 group. Mice in the control group were subcutaneously injected with 0.1 mL of sesame oil daily, in addition to intraperitoneal saline. Mice in the control group were subcutaneously injected with 0.1 mL of sesame oil daily, in addition to intraperitoneal saline. During the entire experiment, the mice were kept in a controlled environment with a temperature of 21 ± 1 °C, relative humidity of 50% ± 5%, a 12-h light/dark cycle, and water and standard rodent food available at all times. With the permission of Changzhi Medical College's Experimental Animal Care & Use Committee (approval number DW2024131). All procedures were carried out in strict compliance with institutional regulations governing the care and utilization of laboratory animals, adhering to the principles of the 3Rs (Replacement, Reduction, Refinement). The study followed the ARRIVE criteria on how to report animal studies.

### 2.4 Determination of the murine estrous cycle

Vaginal smear cytology, which includes washing with physiological saline, was used to obtain vaginal lavage fluid from the selected mice every day for eight consecutive days, starting on the 15th day of the experiment. After Giemsa staining, the samples were examined under a light microscope. The phases of the estrous cycle were delineated as follows: proestrus, primarily characterized by epithelial cells containing nuclei; estrus, dominated by nucleated, keratinized squamous epithelial cells; diestrus, where leukocytes were predominant; and metestrus, during which both keratinized cells and leukocytes were present (Liu Y. et al., 2024).

### 2.5 Sample collection and preprocessing

Before blood samples were collected from the orbital venous plexus, all mice were fasted for 12 h and allowed to drink as much water as desired. We centrifuged the blood samples to separate the serum, which was stored at 80 °C for later analysis. After blood collection, the mice were euthanized by cervical dislocation, and their ovaries and uteri were immediately removed. For histological examination, one side of the ovarian and uterine tissues was fixed in 4% paraformaldehyde, while the other side was quickly frozen in liquid nitrogen and stored at 80 °C for later RNA extraction.

### 2.6 H&E staining procedure for sections of mouse ovarian tissue

Mouse ovarian and uterine tissues were fixed in 4% paraformaldehyde for 24 h, dehydrated, and embedded in paraffin. To assess the histological features of the uterus and morphological development of ovarian follicles, tissue slices were cut to a thickness of 6 μm, stained with hematoxylin and eosin (H&E), and observed under an optical microscope.

### 2.7 Methods for determining the biochemical indicators

A glucometer was used to evaluate fasting blood glucose (FBG) levels, and an enzyme-linked immunosorbent assay (ELISA) was used to measure fasting insulin (FINS) levels. We calculated the homeostasis model evaluation of insulin resistance (HOMA-IR) and insulin sensitivity index (ISI) using the following formulas: HOMA-IR = (FG × FINS)/22.5 and ISI = ln[1/(FINS × FBG)] (Li T. et al., 2021; Zhang et al., 2020). We used a fully automated microplate reader to measure the levels of glutathione (GSH), superoxide dismutase (SOD), and malondialdehyde (MDA) in uterine tissues. We followed the manufacturer's instructions after collecting ileal tissue supernatants.

### 2.8 Evaluation of serum hormone levels

Following the manufacturer's instructions, we used ELISA kits to measure the levels of testosterone (T), estrogen (E), progesterone (PROG), follicle-stimulating hormone (FSH), and luteinizing hormone (LH) in the serum of mice. After the samples were added, the plates were incubated at 37 °C for 1 h and then washed thrice with PBS. The enzyme conjugate was then added, and the plates were kept in the dark at 37 °C for 45 min. After the washing step, TMB substrate was added and kept at 37 °C or 10 min in the dark to allow the color to develop. The addition of 2 M sulfuric acid halted the process. We used blank wells to calibrate the zero point and measured the optical density (OD) at 450 nm. Standard curves were used to determine sample concentrations.

### 2.9 Evaluation of serum, ovarian, and uterine inflammatory biomarkers

We used enzyme-linked immunosorbent assays (ELISA) to measure the levels of IL-4, IL-6, IL-10, and TNF-α in blood, ovarian, and uterine supernatants. The plates were incubated at 37 °C for 45 min after the samples were added. The plates were then cleaned thrice with phosphate-buffered saline (PBS). Thereafter, an enzyme conjugate was added, and the plates were kept in the dark at 37 °C for 30 min. The washing process was performed twice. Thereafter, the TMB substrate was added, and the color was developed in the dark at 37 °C for 15 min. The reaction was stopped by adding 200 μL of stop solution. The optical density (OD) was measured at 450 nm using a blank well as a reference. We determined the sample concentrations using standard curves.

### 2.10 Quantitative real-time PCR (qRT-PCR)

Total RNA was extracted from ovarian and uterine tissues using TRIzol^®^ reagent in accordance with the manufacturer's instructions. The specific steps were as follows: The TRIzol technique was used to extract total RNA. Briefly, cell samples were collected and homogenized in TRIzol reagent on ice, or tissue samples were snap-frozen in liquid nitrogen and pulverized. RNA was precipitated with isopropanol, cleaned with 75% ethanol, and dissolved in RNase-free water after the aqueous phase was separated using chloroform. The integrity of RNA was confirmed through agarose gel electrophoresis, which revealed distinct 28S and 18S rRNA bands with an approximate ratio of 2:1. The RNA concentration and purity were determined using NanoDrop (A260/A280 ratio 1.9–2.1). The reaction was terminated with EDTA or column purification was performed directly, and RNA quality was reassessed to ensure the absence of genomic DNA contamination. RNA samples were treated with DNase at 37 °C for 15–20 min. Using a reverse transcription kit with either random primers or oligo (dT) primers, 1 μg of total RNA was reverse transcribed for cDNA synthesis. The enzyme was inactivated at 85 °C for 5 min, primer annealing was conducted at 25 °C for 10 min, and reverse transcription was conducted at 37 °C for 120 min. The reaction conditions were as follows. The cDNA products were aliquoted and stored at −20 °C.

Real-time quantitative PCR (qPCR) experimental procedures: We used real-time quantitative PCR (qPCR) to evaluate the levels of Cyp11A, Cyp17A, Cyp19A, VEGFA, VEGFAR, MMP2, MMP9, TIMP1, and TIMP2. The reactions were performed in a 20 μL volume containing SYBR Green I Master Mix, 0.5 μM forward and reverse primers, and 2 μL of cDNA template. The thermal cycling conditions included a 10-min denaturation step at 95 °C, followed by 40 cycles at 95 °C for 15 s and 60 °C for 1 min. Melting curve analysis confirmed the specificity of the primers. We utilized the 2 ^−ΔΔCt^ method to determine the expression levels of each gene, with β-actin as the internal reference for normalization. Supplementary Table 1 shows the primer sequences for each target gene.

### 2.11 Metabolic profiling of bile acid in fecal

We developed a targeted UPLC-MS/MS technique with internal standard calibration to measure 33 bile acids simultaneously (22 free and 11 conjugated). We used eight deuterated bile acids (d4-CA, d4-CDCA, d4-DCA, d4-LCA, d4-UDCA, d4-GCA, d4-GCDCA, and d4-TCA) as internal standards at a concentration of 100 ng/mL. Sample Preparation: Fecal samples (50 mg) were extracted using ice-cold 80% methanol (500 μL) containing internal standards, homogenized using bead beating (50 Hz, 3 min), vortexed for 20 min, and centrifuged (20,000 × g, 4 °C). After incubating at 30 °C for 1 h, the supernatant (60 μL) was derivatized with 3-NPH (200 mM) and EDC (120 mM). It was then centrifuged and analyzed using UPLC-MS/MS. Conditions for UPLC-MS/MS: The ACQUITY BEH C18 column (100 mm × 2.1 mm, 1.7 μm) was used for chromatographic separation. Mobile phase A (0.1% formic acid in water) and B (0.1% formic acid in acetonitrile) were used for gradient elusion at 0.4 mL/min at 45 °C. We used a Xeno TQ-S triple quadrupole mass spectrometer in the ESI? MRM mode for mass spectrometry. The capillary voltage was −4.5 kV, and the source temperature at 350 °C. Calibration and Quality Control: Nine-point calibration curves (0.1–1,000 ng/mL) with *R*^2^ > 0.995; the lower limit of quantification (LLOQ) was defined as S/N ≥ 10 with an accuracy of ±20%. Each batch had low, medium, and high QC samples (with an accuracy of ±15% and a CV of < 15%), as well as procedural and derivatization blanks. Data Analysis: Quantification was performed using the internal standard approach via Mass Lynx v4.1; statistical significance was assessed using one-way ANOVA with Tukey's *post hoc* test. Multiple testing correction was performed using the Benjamini-Hochberg false discovery rate (FDR) method across all bile acids. FDR-adjusted *P-values* (q values) < 0.10 were considered statistically significant. The data are presented as the mean plus or minus the standard error of the mean (Qi et al., 2019).

### 2.12 Analysis of fecal microbiome sequencing

The bioinformatics workflow for gut microbiota analysis followed established techniques, as previously described (Esfandiarinezhad et al., 2025). Fecal samples were gathered in sterile containers, rapidly frozen in liquid nitrogen, and preserved at −80 °C until further processing. We used the QIAamp PowerFecal Pro DNA Kit (Qiagen) to extract microbial DNA, following the instructions that came with the kit. We used NanoDrop (A260/A280 ratio 1.8–2.0) and agarose gel electrophoresis to check the DNA's content and purity. Using the universal primers 341F (5′-CCTACGGGNGGCWGCAG-3′) and 806R (5′-GGACTACHVGGGTATCTAAT-3′) with Illumina adapter sequences, the V3-V4 hypervariable region of the bacterial 16S rRNA gene was amplified. The PCR reactions were 50 μL and comprised 25 μL of 2 × Phusion High-Fidelity PCR Master Mix, 1 μL of each primer (10 μM), and 50 ng of template DNA. The temperature changed from 98 °C for 30 s to 25 °C for 10 s, then from 55 °C for 30 s to 72 °C for 30 s. Then, it stayed at 72 °C for 5 min. We used AM Pure XP beads to clean up the amplicons, and then we did a second PCR to add dual-index barcodes. We used Qubit to measure the final libraries and then mixed them together in equal amounts so that we could do paired-end sequencing (2 × 250 bp) on the Illumina NovaSeq platform. We used QIIME2 (v2023.2) to demultiplex and filter the quality of the raw reads. Using the DADA2 plugin, chimeric sequences were taken out, and amplicon sequence variants (ASVs) were made. The SILVA database (v138) was used to do taxonomic categorization with a trained naive Bayes classifier. We figured out the α-diversity indices (Chao1, Shannon, and Simpson) and used principal coordinate analysis (PCoA) based on Bray-Curtis and weighted UniFrac distances to figure out the β-diversity. We utilized PERMANOVA to see if there were any differences between the groups. We used LEfSe (LDA score >4.0) and DESeq2 with false discovery rate (FDR) correction (*q* < 0.05) to find taxa that were differentially abundant. Quality Control and Validation: The A260/A280 ratio for DNA extraction had to be between 1.8 and 2.0, and the color had to be normal. PCR amplification produced a singular, distinct band devoid of primer dimers, as validated by 2% agarose gel electrophoresis. Each batch had two negative controls (an extraction blank and a no-template control) that showed no detectable amplification (Ct > 38) and a positive control that used the Escherichia coli standard strain to check the experimental setup. Using fast (v0.23.2) with the following thresholds, we got quality-filtered reads: a minimum Phred quality score of Q20, a minimum length of 50 bp, and the removal of reads with more than 10% ambiguous bases. The average sequencing depth was 105,432 ± 23,156 reads per sample (range: 52,891–148,763). Rarefaction curves leveled off around 30,000 reads, which showed that there was enough coverage. The Benjamini-Hochberg method (*q* < 0.05) was used to control for false discovery rate (FDR) in the analysis of differential abundance.

### 2.13 Statistical analysis

Before the analysis, all samples were assigned new labels, and an independent investigator maintained the coding key. The statistician obtained datasets that did not contain any identifying information during the data analysis step. After the experiments were conducted, the coding key was compared with the original data for the last step of the statistical analysis. All statistical analyses were performed using biological replicates. We used SPSS 22.0 to analyze the data, and the results are shown as the mean ± standard error of the mean (mean ± SEM), with *n* values indicating the number of independent mice. GraphPad Prism 8.0 was used to prepare the figures. One-way analysis of variance (ANOVA), followed by Tukey's *post hoc* test or an independent two-sample *t*-test, was used to compare the groups. In experiments with technical replicates (such as qPCR or histological quantification), the technical replicates from the same sample were averaged to obtain one data point for statistical analysis. The *n* values represent the biological replicates. Statistical significance is indicated as follows: ^*^P < 0.05, ^**^P < 0.01, and ^ns^*P* > 0.05.

## 3 Results

### 3.1 Lobetyolin's effects on DHEA mice's body weight and blood glucose levels in mice

**Figure 1:**
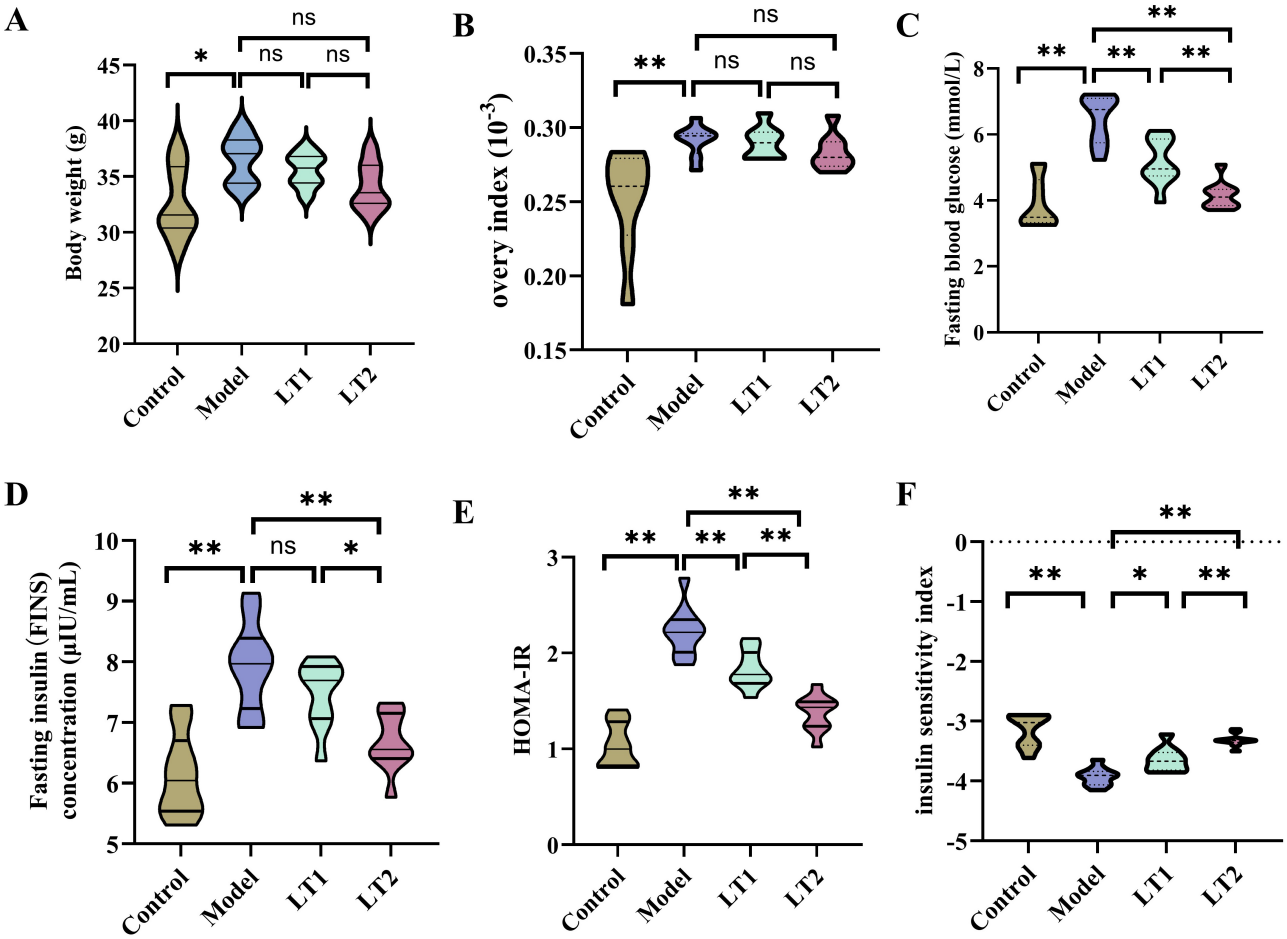
Effect of lobetyolin on body weight and blood sugar levels of mice treated with DHEA. *n* = 10. **(A)** Body weights of mice. **(B)** Ovarian index. **(C)** Fasting blood sugar (FBG) levels. **(D)** Fasting insulin (FINS) levels. **(E)** HOMA-IR. **(F)** ISI. Quantified values are expressed as the mean ± SEM in *n* = 10 biologically independent samples. *P < 0.05, **P < 0.01, and ^ns^*P* > 0.05.

As depicted Figure 1, the Model group exhibited significantly elevated body weight (Figure 1A) and ovarian index (Figure 1B) compared to the Control group. After intervention with lobetyolin, body weight and ovarian index decreased in a dose-dependent manner in the LT1 and LT2 groups. The Model group exhibited significantly higher fasting blood glucose levels (Figure 1C), HOMA-IR (Figure 1D) and FINS (Figure 1E), and ISI (Figure 1F) than that of the Control group. After LT intervention, the levels of FBG, HOMA-IR, FINS, and ISI showed a dose-dependent reduction in the LT1 and LT2 groups, which were considerably lower than those in the Model group. These results indicate that lobetyolin efficiently mitigates DHEA-induced glucose metabolic disturbances in mice, underscoring its potential significance in regulating metabolic syndrome.

### 3.2 Lobetyolin's effects on DHEA mice's ovarian blood supply, tissue structure, and estrous cycle

**Figure 2:**
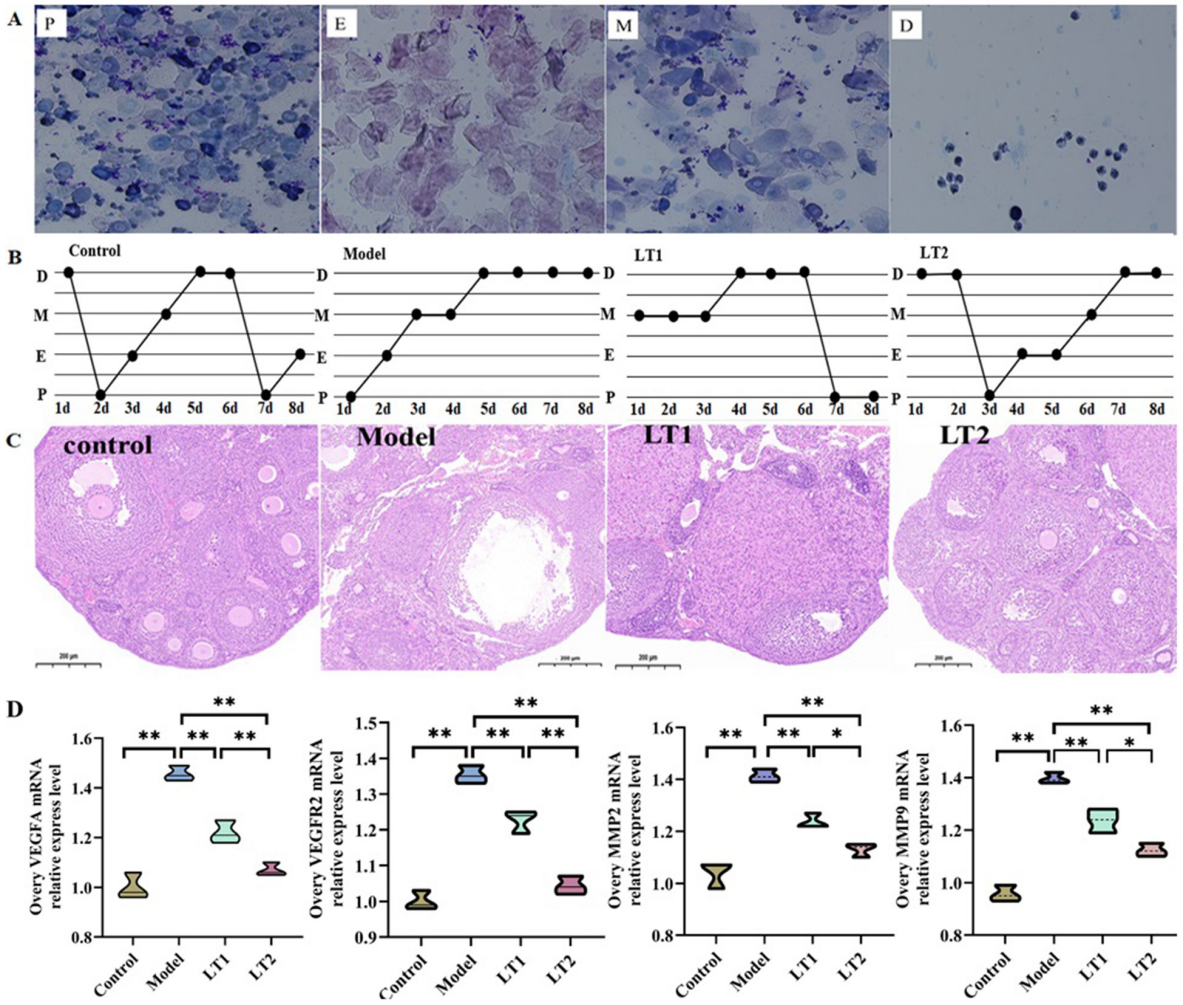
Effects of lobetyolin on estrous cycle, ovarian tissue structure, and ovarian blood supply in DHEA mice. **(A)** Representative images of mouse vaginal smears after Wright-Giemsa staining. P, E, M, and D indicate proestrus, estrus, metestrus, and *n* = 5 biologically independent samples. **(B)** Modifications in the mouse estrous cycle in *n* = 5 biologically independent samples. **(C)** Histological slices of mouse ovarian tissue (H&E staining, × 100 magnification, scale bar = 200 μm), *n* = 3 biologically independent samples. **(D)** Variations in the expression levels of VEGFA, VEGFR2, MMP2, and MMP9 in mouse ovarian tissues, *n* = 3 biologically independent samples. Quantified data are displayed as mean ± SEM. *P < 0.05, **P < 0.01.

Vaginal smear tests were performed every 8 days (Figures 2A, B). After LT intervention, both the LT1 and LT2 groups returned to fairly regular rhythmic patterns. This indicates that LT corrected the DHEA-induced estrous cycle dysregulation in mice. This indicates that LT corrected the DHEA-induced estrous cycle dysregulation in mice. This indicates that LT corrected the DHEA-induced estrous cycle dysregulation in mice.

Figure 2C shows a considerable prevalence of cystically dilated and atretic follicles in the model group, and the granulosa cell layers in these follicles were either thinner or absent, with no oocytes present, which is expected in PCOS mice. In the Control group, oocytes and the corona radiata were discernible within mature follicles, granulosa cells were arranged in 8–9 structured layers, and several corpora lutea were identified in mature follicles. In the LT1 and LT2 groups that received LT intervention, the granulosa cell layers were of intermediate quantity, and mature follicles containing oocytes were observed in the interstitial tissue of the ovaries.

Figure 2D shows that the levels of VEGFA, VEGFR, MMP2, and MMP9 were much higher in the ovaries of the Model group than in the Control group. This suggests that the pathology of the Model may involve excessive activation of angiogenic pathways. After LT intervention, the expression of these genes was downregulated in a dose-dependent manner in the LT1 and LT2 groups, with more significant suppression in the LT2 group (*P* < 0.05). These findings suggest that LT may mitigate DHEA-induced ovarian vascular anomalies by suppressing angiogenesis.

### 3.3 Lobetyolin's effect on ovarian-related genes and serum hormones in DHEA mice

**Figure 3:**
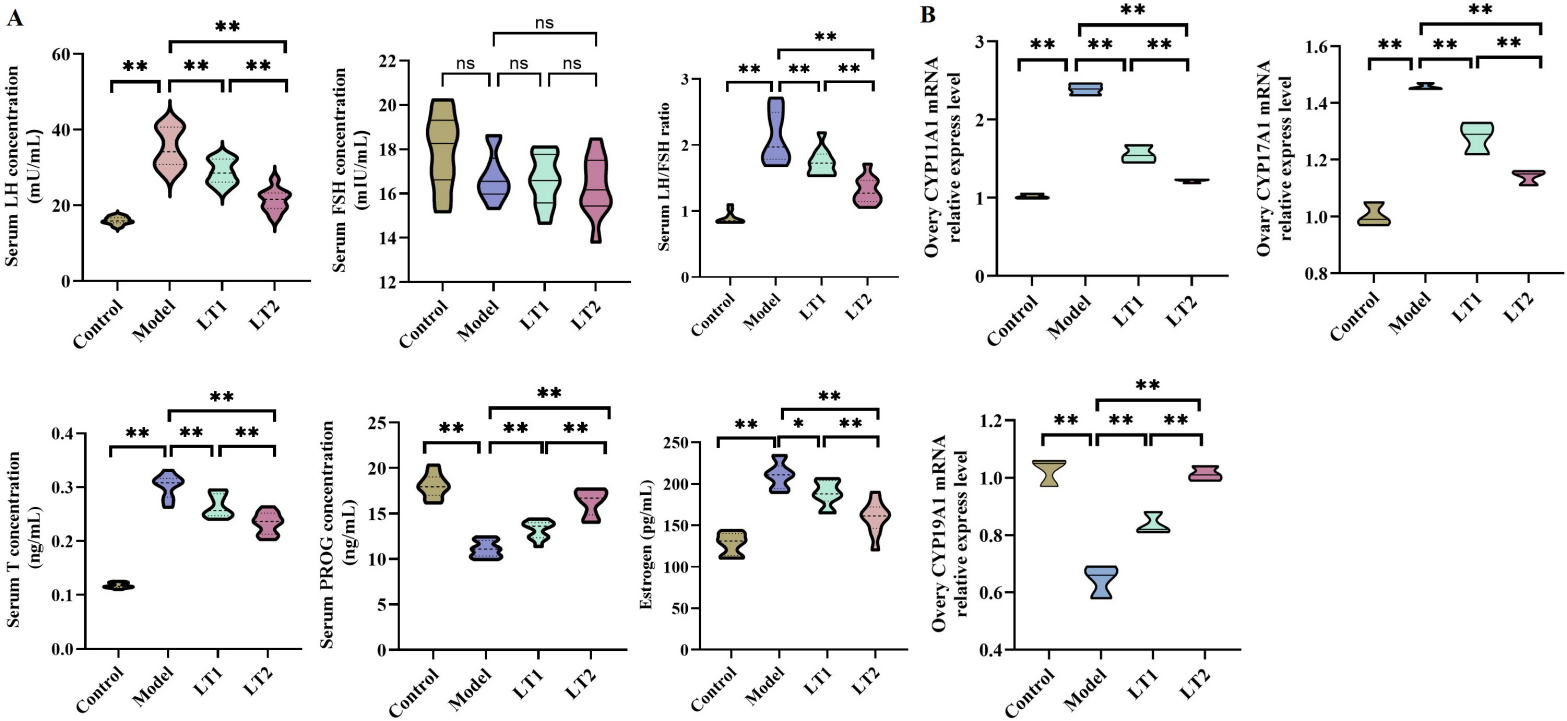
Effect of lobetyin on ovarian-related genes and serum hormones in DHEA-treated mice. **(A)** Sex hormone levels in mice (*n* = 10 biologically independent samples). **(B)** CYP11A1, CYP17A1, and CYP19A1 gene expression levels in mouse ovarian tissue (*n* = 3 biologically independent samples). Mean ± SEM was used to display quantitative data. *P < 0.05, **P < 0.01, and ^ns^*P* > 0.05.

Figure 3A shows that the serum levels of LH, the LH/FSH ratio, T, and E in the Model group were considerably higher than those in the Control group, whereas PROG levels were significantly lower in the Model group. Following LT intervention, the LT1 and LT2 groups showed a dose-dependent decrease in T, LH, the LH/FSH ratio, and E, with the LT2 group having a stronger effect than the model group. PROG levels increased significantly with increasing LT concentrations. These findings suggest that LT effectively alleviates the DHEA-induced imbalance in sex hormone metabolism in mice by lowering hyperandrogenism (T and LH) and prolactin levels, restoring the gonadotropin ratio (LH/FSH), and increasing progesterone secretion, indicating its potential for regulating hormone disorders.

Figure 3B shows that CYP19A1 expression was dramatically reduced in the model group, although the expression levels of CYP11A1 and CYP17A1 in ovarian tissues were much higher than those in the Control group. LT intervention resulted in a dose-dependent elevation of CYP19A1 expression, with the LT2 group showing the most significant increase. In contrast, the expression levels of CYP11A1 and CYP17A1 in the LT1 and LT2 groups decreased in a dose-dependent manner, with the LT2 group displaying more significant inhibition.

These findings imply that LT may alleviate ovarian hormone imbalance by increasing the expression of genes involved in estrogen production (CYP19A1) and reducing the expression of genes involved in androgen synthesis (CYP17A1) and cholesterol metabolism (CYP11A1).

### 3.4 Lobetyolin's effect on inflammatory markers in the serum and ovaries of DHEA mice

**Figure 4:**
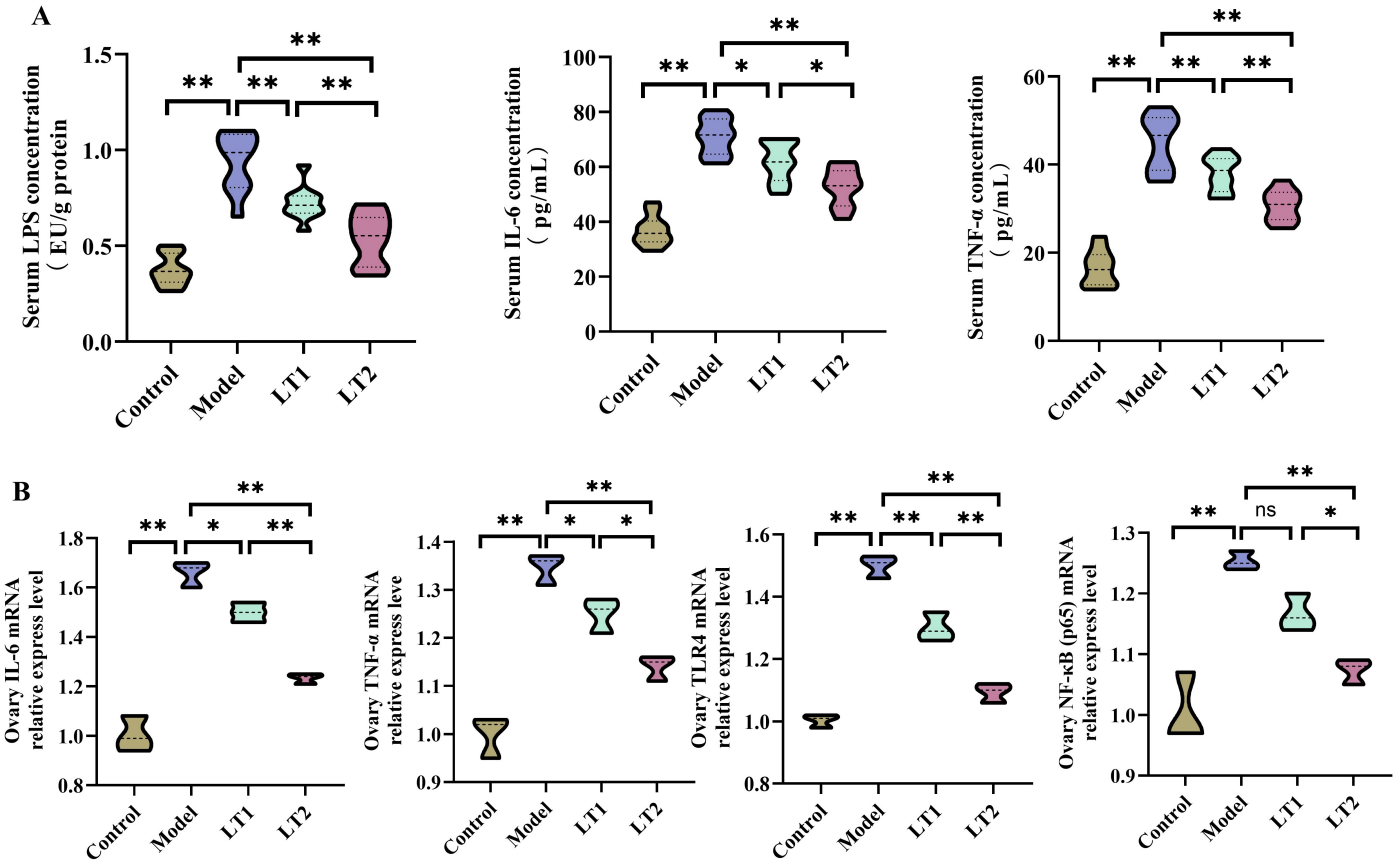
Effects of lobetyolin on inflammatory markers in DHEA mouse serum and ovaries. **(A)** Systemic inflammation in mice (*n* = 3 biologically independent samples). **(B)** Expression levels of inflammation-related genes in mouse ovarian tissue (*n* = 3 biologically independent samples). Quantified data are presented as mean ± SEM. *P < 0.05, **P < 0.01, ^ns^*P* > 0.05.

Figure 4A shows that the serum levels of TNF-α, IL-6, and LPS were markedly increased in the Model group compared to the Control group. In the Model group, the levels of TNF-α, IL-6, TLR4, and NF-κB (p65) in ovarian tissue were much higher than those in the Control group (Figure 4B). This demonstrates that DHEA caused a systemic inflammatory response and activated local ovarian signaling pathways in the ovaries. After LT treatment, elevated levels of TNF-α, IL-6, and LPS in the blood were reduced in a dose-dependent manner in the LT1 and LT2 groups (Figures 4A, B). Despite the small sample size limiting statistical robustness, the effect direction was consistent with changes in serum inflammatory markers. These preliminary findings suggest that LT may exert local anti-inflammatory effects in the ovary, but validation in independent animal experiments with an expanded sample size is required.

### 3.5 Lobetyolin's effect on DHEA mice's uterine oxidative stress and inflammation

**Figure 5:**
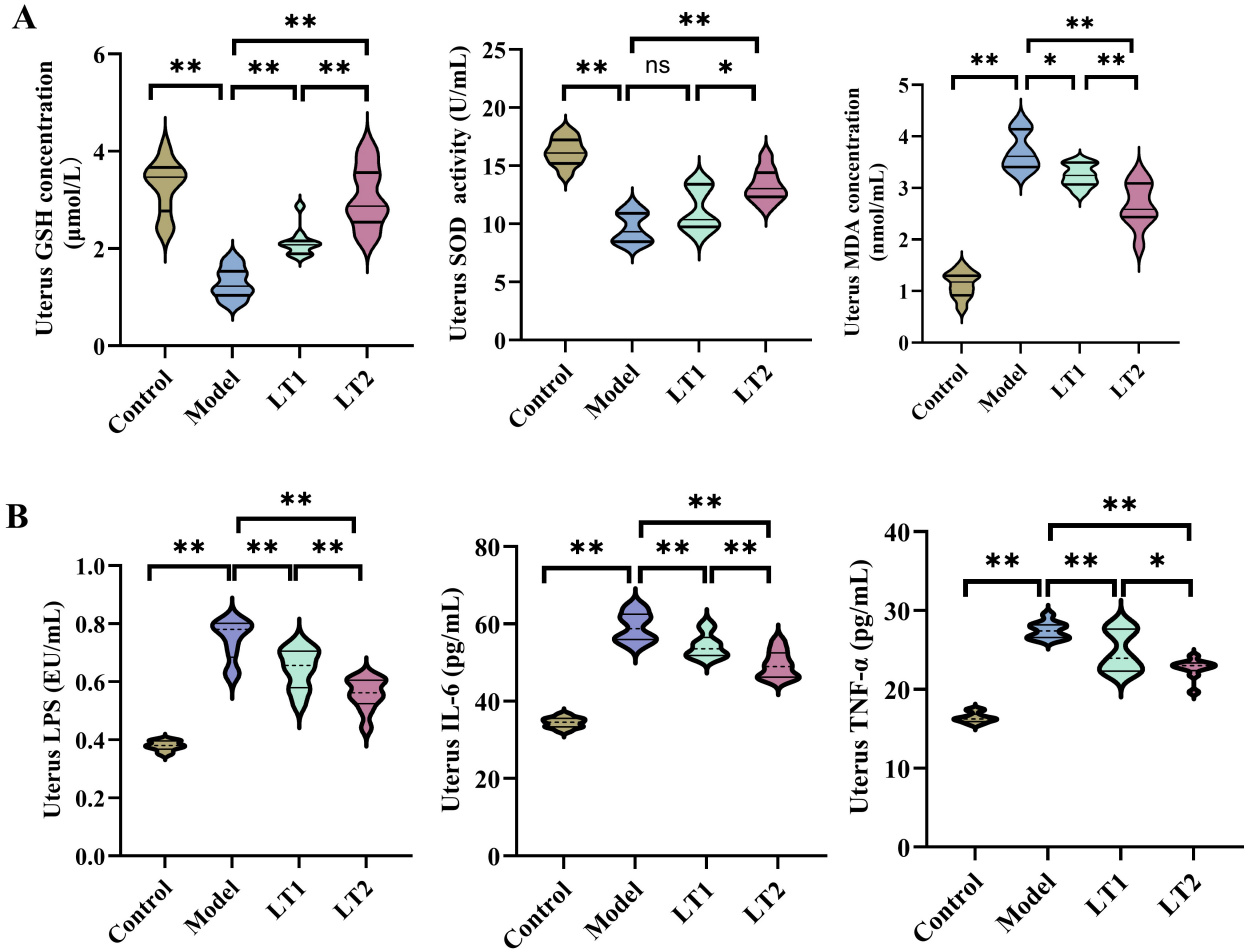
Effect of lobetylin on oxidative stress and inflammatory levels in uterine tissues of DHEA-treated mice. **(A)** Levels of oxidative stress indicators in mouse uterine tissue. **(B**) Concentrations of inflammatory markers in mouse uterine tissue. Quantified values are expressed as the mean ± SEM (*n* = 10 biologically independent samples). *P < 0.05, **P < 0.01, ^ns^*P* > 0.05.

Figure 5A illustrates that, relative to the Control group, MDA levels were significantly higher in the Model group, whereas glutathione (GSH) and superoxide dismutase (SOD) activity levels were significantly lower. MDA levels showed a dose-dependent decrease in the LT1 and LT2 groups, whereas lobetyolin administration resulted in a dose-dependent increase in GSH levels and SOD activity. The levels of LPS, IL-6, and TNF-α in the uterus were considerably higher in the Model group than in the Control group (Figure 5B). Both the LT1 and LT2 groups experienced a dose-dependent decrease in uterine TNF-α, IL-6, and LPS levels after LT intervention, with LT2 exhibiting a more noticeable effect. These findings support the idea that LT reduces the uterine inflammatory response induced by DHEA by suppressing the release of proinflammatory mediators.

### 3.6 Lobetyolin's effect on the extracellular matrix and uterine angiogenesis in DHEA mice

**Figure 6:**
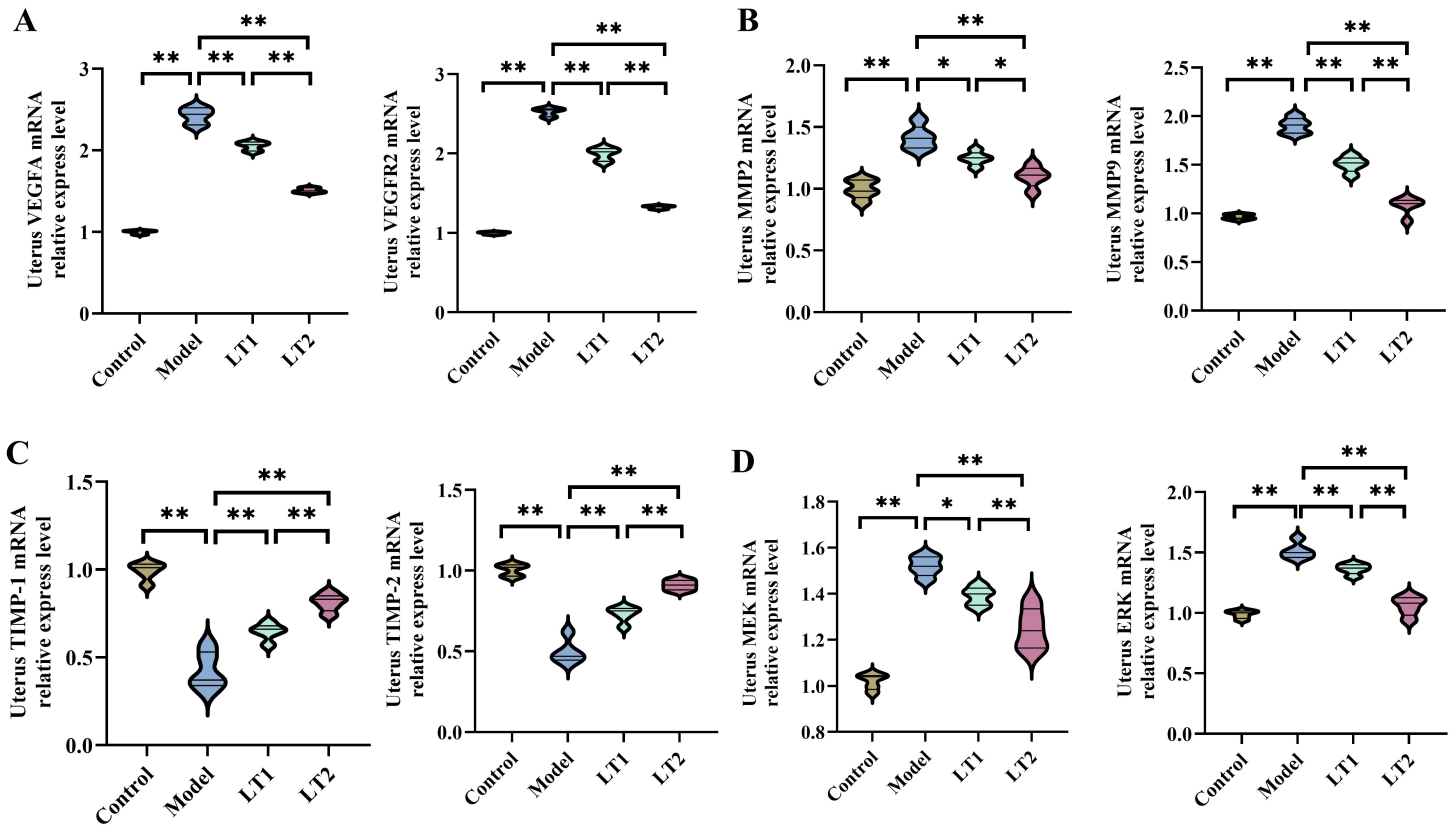
Effect of lobetyolin on angiogenesis and extracellular matrix in uterine tissue of DHEA-induced mice. **(A)** Gene expression levels of VEGFA and VEGFR2 in uterine tissue of mice. **(B)** MMP2 and MMP9 gene expression levels in mouse uterine tissue. **(C)** TIMP1 and TIMP2 gene expression levels in mouse uterine tissue. The quantified results are presented as mean ± SEM, *n* = 3 biologically independent samples. **(D)** Shows that the mRNA levels of MEK and ERK in the uterine tissue of mice in the Model group were much higher than those in the Control group. After LT intervention, the LT1 and LT2 groups showed a dose-dependent decrease in the levels of MMP9 and MMP2 mRNA in the uterus. *P < 0.05, **P < 0.01, ^ns^*P* > 0.05.

In the Model group, the mRNA levels of VEGFA and VEGFR2 in the uterine tissue of mice were much higher than those in the Control group (Figure 6A). Following LT intervention, uterine mRNA levels of VEGFA and VEGFR2 decreased in a dose-dependent manner following LT interventions. Figure 6B shows that the mRNA levels of MMP9 and MMP2 in the uterine tissue of mice in the Model group were much higher than those in the Control group. After LT intervention, the LT1 and LT2 groups showed a dose-dependent decrease in the levels of MMP9 and MMP2 mRNA in the uterus. In contrast, the mRNA expression levels of TIMP1 and TIMP2 in the uterine tissue of mice in the Model group were much lower than those in the Control group (Figure 6C). In the LT1 and LT2 groups, the uterine mRNA levels of TIMP1 and TIMP2 exhibited a dose-dependent increase after the LT intervention. Figure 6D shows that the mRNA levels of MEK and ERK in the uterine tissue of mice in the Model group were much higher than those in the Control group. After LT intervention, the LT1 and LT2 groups showed a dose-dependent decrease in the levels of MMP9 and MMP2 mRNA in the uterus. These findings indicate that LT administration markedly ameliorated aberrant expression profiles of angiogenic factors, ECM remodeling indicators, and MAPK pathway components in the uterus of PCOS rats, exhibiting a dose-responsive relationship between LT1 and LT2 treatments.

### 3.7 Lobetyolin's effect on DHEA mice's metabolism of fecal bile acid

**Figure 7:**
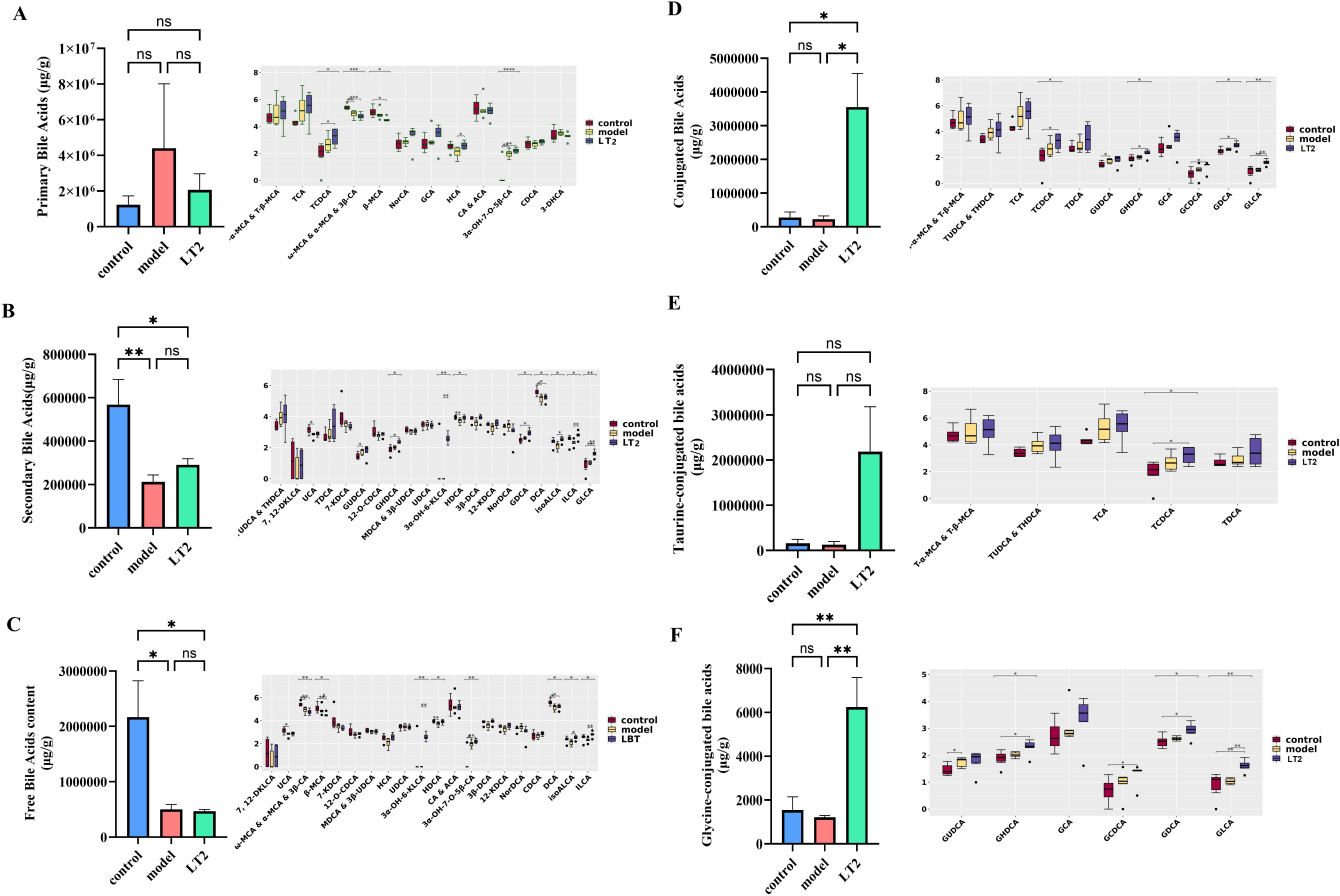
Effect of lobetyolin on fecal bile acid metabolism in DHEA-induced mice. **(A)** Primary bile acid levels in mouse feces. **(B)** Levels of secondary bile acids in mouse feces. **(C)** Amount of free bile acids in mouse feces. **(D)** Levels of conjugated bile acids in mouse feces. **(E)** Taurine-conjugated bile acid levels in mouse feces. **(F)** Glycine-conjugated bile acid levels in mouse feces. Quantified data are displayed as mean ± SEM, *n* = 6 biologically independent samples. *P < 0.05, **P < 0.01, ^ns^*P* > 0.05.

The Model group mice exhibited pronounced bile acid metabolic abnormalities compared to the Control group, as indicated by the bile acid metabolism study (Figures 7A–F). The Model group showed a significant drop in ω/α/3β-MCA and a significant increase in 3-α-OH-7-O-5β-CA levels, which are two of the most important bile acids (Figure 7A). The decrease in UCA, HDCA, and DCA levels indicated a significant reduction in the total amount of secondary bile acids dropped significantly (Figure 7B). The overall amount of free bile acids decreased as the mixture changed (Figure 7C). Simultaneously, the levels of secondary bile acid components, including 3α-OH-6-KLCA, isoALCA, and ILCA, increased significantly (Figure 7B). As shown in Table 1, GLCA, GHDCA, and GDCA were all considerably higher in the LT2 group than in the Model group. The bile acid metabolic profile also showed significant increases in important components, such as TCDCA, GHDCA, GCDCA, GDCA, and GLCA (Figures 7D–F). Simultaneously, the levels of secondary bile acid components, including 3α-OH-6-KLCA, isoALCA, and ILCA, increased significantly (Figure 7B). The LT2 group exhibited markedly increased levels of HCA and TCDCA, but there was no statistically significant variation in the overall quantities of primary or taurine-conjugated bile acids across the three groups. These results indicated that LT2 may alleviate DHEA-induced bile acid metabolic dysregulation in DHEA mice by enhancing bile acid conjugation, particularly the glycine conjugation pathway.

**Table 1:** Comparison of glycine-conjugated bile acid levels.

Group	GHDCA	GCDCA	GDCA	GLCA	GCA	GUDCA
Control	85.82 ± 19.63^Bb^	6.31 ± 2.67^b^	365.50 ± 82.86^b^	9.92 ± 2.89^B^	1,045 ± 554.1	31.18 ± 6.99^b^
Model	104.70 ± 9.79^ABb^	12.88 ± 4.93^ab^	420.20 ± 28.38^b^	10.10 ± 1.12^B^	4,898 ± 4261	57.92 ± 8.85^ab^
LT2	226.40 ± 44.56^Aa^	23.72 ± 4.53^a^	989.20 ± 235.10^a^	43.90 ± 8.88^A^	5,238 ± 1,948	76.35 ± 17.28^a^

Different lowercase letters in the same column indicate significant differences, while different uppercase letters indicate extremely significant differences.

### 3.8 Lobetyolin's effect on DHEA mice's fecal microbiota diversity

**Figure 8:**
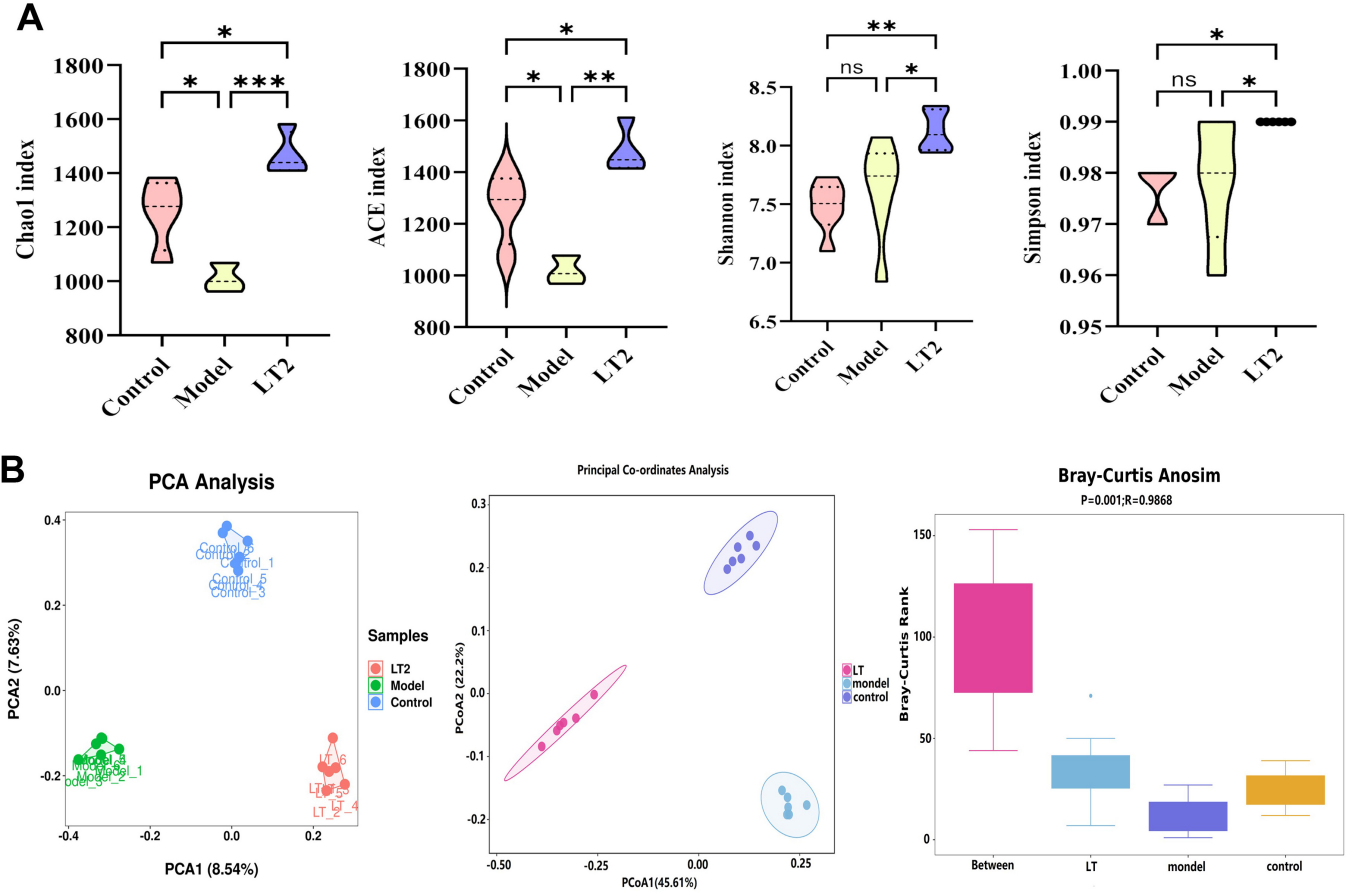
Effect of LT on fecal microbiota diversity in DHEA-induced PCOS mice. **(A)** Fecal microbiota alpha diversity. **(B)** Fecal microbiota beta diversity. Mean ± SEM was used to display quantitative data for *n* = 6 biologically independent samples. *P < 0.05, **P < 0.01, ***P < 0.001, ^ns^*P* > 0.05.

Substantial dysbiosis was detected in the Model group using gut microbial diversity profiling. The alpha diversity indicators, Chao1 and ACE, which quantify the number of different species, were much lower in the Model group (*P* < 0.05) than in the Control group (Figure 8A). This shows that there were fewer microbial species in the Model group. After the LT2 intervention, the Chao1 and ACE indices increased to levels much greater than those in the Model group (*P* < 0.05). Simultaneously, the Simpson (dominance) and Shannon (species diversity) indices increased. This suggests that LT2 makes the gut microbiota more diverse and richer. The microbiota compositions of the Control, Model, and LT2 groups were distinctly different, as shown by the three-dimensional PCA/PCoA clustering diagram utilizing beta diversity analysis (Figure 8B). Bray-Curti's dissimilarity testing (ANOSIM) (*R* = 0.9868, *P* = 0.001) also showed substantial differences, indicating that DHEA disrupts the balance of the intestinal microecology, while LT can reverse this imbalance, which is a symptom of abnormal regulation in PCOS. These results suggest that LT2 may alleviate disturbances by restoring gut microbiota diversity.

### 3.9 Lobetyolin's effect on mouse phyla and species

**Figure 9:**
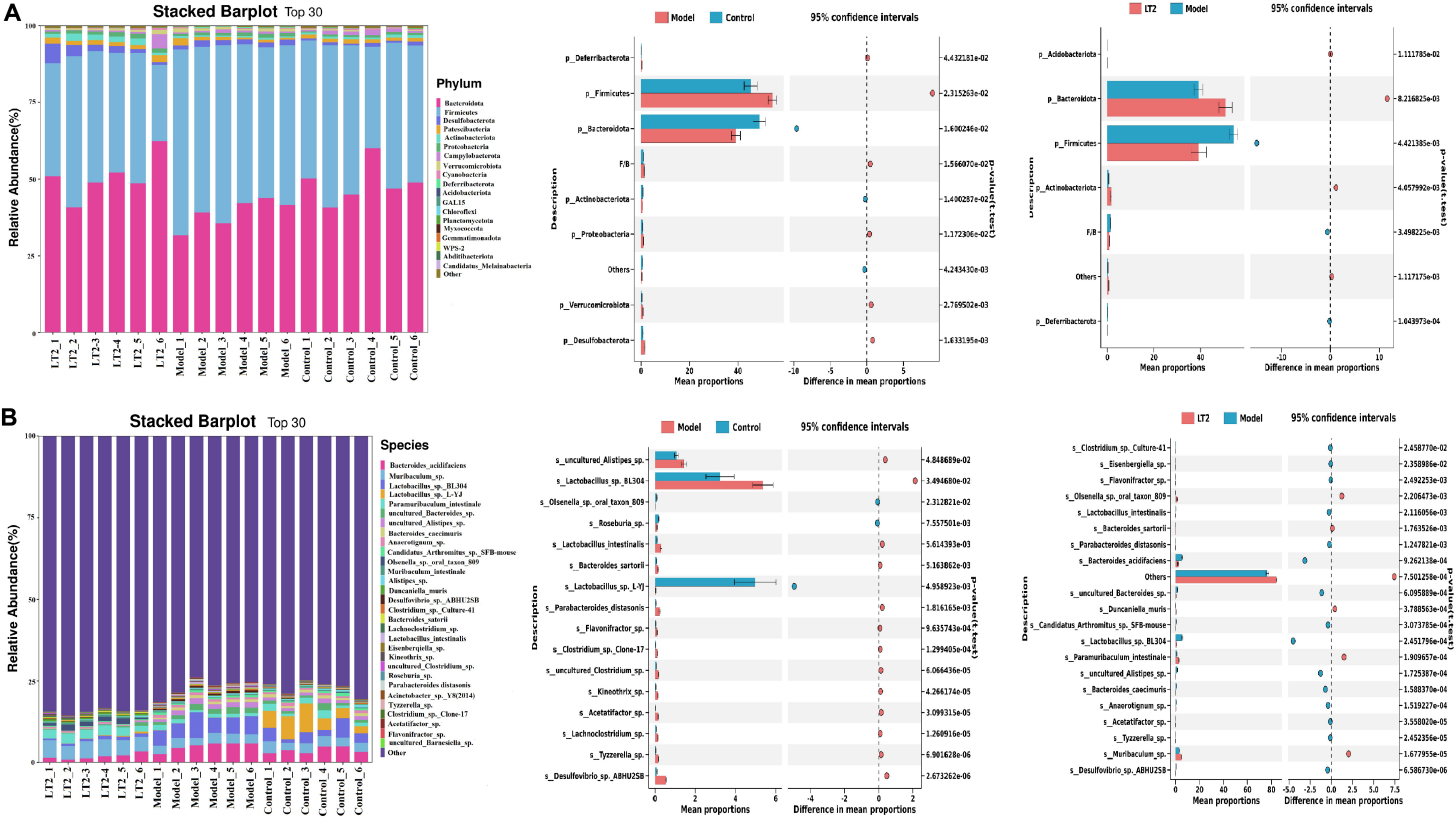
Effect of lobetyolin on fecal microbiota at the phylum and species levels in DHEA-treated mice. **(A)** Phylum-level composition of fecal microbiota; **(B)** Species-level composition of fecal microbiota. Quantified data are expressed as the mean ± SEM (*n* = 6 biologically independent samples).

Figure 9 illustrates the outcomes of the analysis of fecal microbial phyla and species in mice. Figure 9A shows the top 20 identified phyla. *Firmicutes* and *Bacteroidetes* constituted most of the core microbiota, accounting for 90% of the entire microbiota. Notably, phylum-level dysbiosis was detected in the model group relative to the control group: the abundances of *Verrucomicrobiota* and *Deferribacterota* dramatically increased (*P* < 0.05), whereas the abundance of *Bacteroidetes* significantly decreased (*P* < 0.05). The *Firmicutes*/*Bacteroidetes* (F/B) ratio was significantly increased (*P* < 0.05). The LT2 group had significantly more *Bacteroidetes* and *Actinobacterota* (*P* < 0.05) than the Model group. In contrast, the LT2 group had significantly lower abundances of *Firmicutes* and *Deferribacterota* and a lower F/B ratio (*P* < 0.05). These results indicate that DEHA induces intestinal dysbiosis in mice. A high F/B ratio is associated with an increased risk of metabolic problems in the host, as it is usually associated with an inflammatory state. LT2 corrected the F/B ratio imbalance in the Model group by lowering the number of *Firmicutes* and increasing the number of *Bacteroidetes*.

Figure 9B illustrates differences at the species level. The levels of *s_Lactob_L-YJ* decreased (*P* < 0.05), but the model group had much higher levels of *s_uncultured_Alistipes_sp*. and *s_Lactobacillus_sp than the YJ group._BL304* (*P* < 0.05) than that of the control group. Commensal bacteria, including *s_Muribaculum_sp., s_Paramuribaculum_intestinale*, and *s_Olsenella_sp._oral_Taxon_809*, exhibited considerable proliferation throughout microbial species remodeling in the LT2 group (*P* < 0.05). Simultaneously, species, such as *s_Bacteroides_acidifaciens, s_Lactob_BL304*, and *s_uncultured_Alistipes_sp*., showed a significant decrease in abundance (*P* < 0.05). These results indicate that LT may modulate lipid metabolism and exhibit anti-inflammatory properties by elevating the levels of *s_Muribaculum_sp*. and *s_Paramuribaculum_intestinale* while reducing the levels of *s_Bacteroides_acidifaciens*.

### 3.10 Lobetyolin's effect on mice's fecal signature microbiota

**Figure 10:**
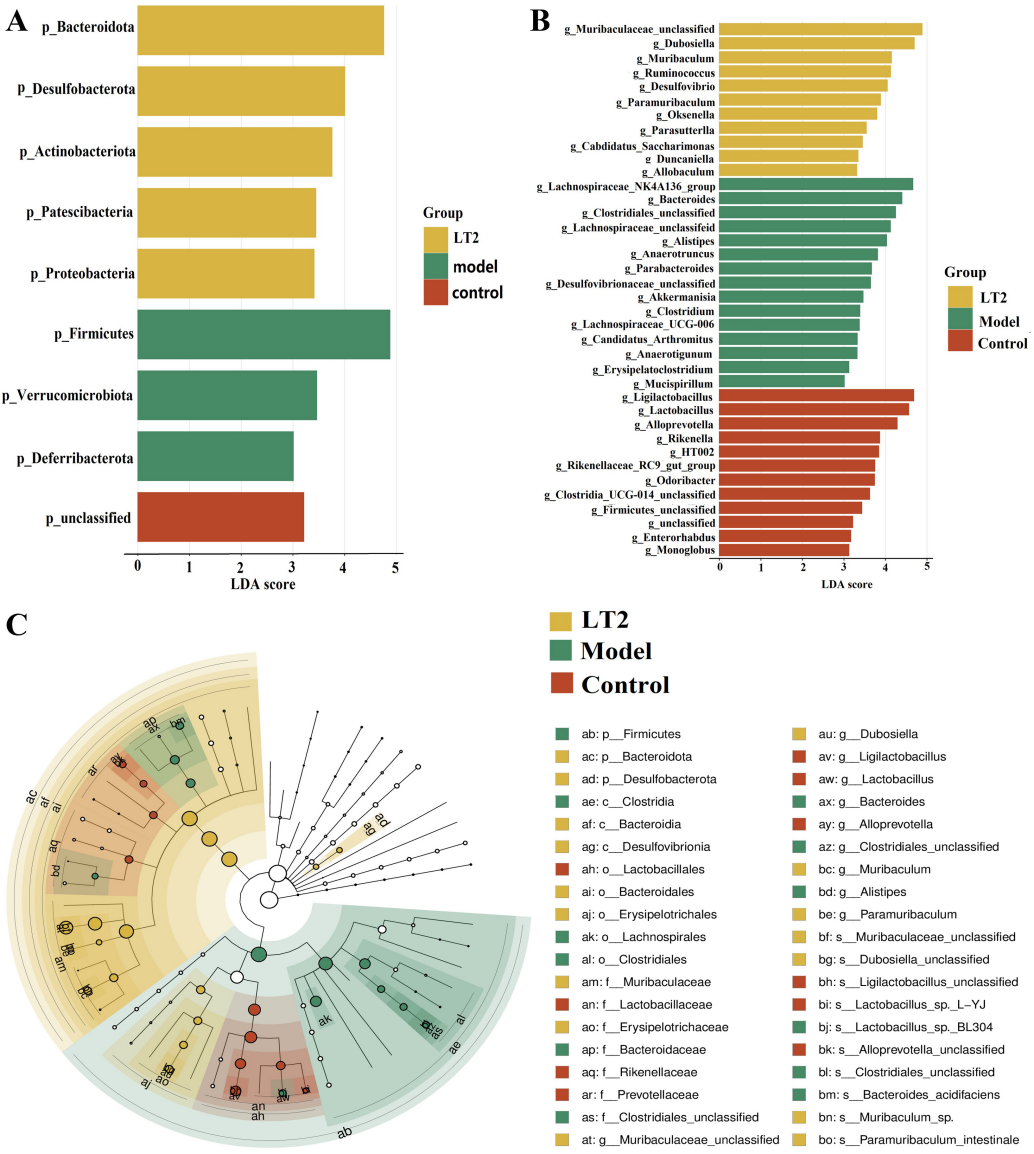
Effect of lobetyolin on the distinctive fecal microbiota of mice administered dehydroepiandrosterone. **(A)** Unique bacterial phyla found in mouse feces; **(B)** Distinct bacterial genera in murine feces; **(C)** Phylogenetic cladogram of fecal microbial species in mice. Quantified data are expressed as mean ± SEM, *n* = 6 biologically independent samples.

Linear discriminant analysis (LDA) indicated that LDA ≥ 4 revealed that the Firmicutes phylum was the signature phylum in the model group, whereas the Bacteroidetes phyla were the signature phyla in the LT2 group (Figure 10A). The Control group had *Alloprevotella, Lactobacillus*, and *Ligilactobacillus* as the signature genera (Figure 10B). The most common genera in the model group were *g_Lachnospiraceae_NK4A136_group, g_Bacteroides, g_Clostridiales_unclassified, g_Lachnospiraceae_unclassified*, and *g_Alistipes*. The main groups in the LT2 group were *g_Muribaculaceae_unclassified, g_Dubosiella, and g_Muribaculum*. Figure 10C shows that groups such as *Dubosiella, Muribaculaceae_unclassified*, and *Muribaculum* were close to each other. *Dubosiella*, a genus clearly linked to the LT2 group, can be used as a biomarker for the effects of an intervention. On the other hand, taxa like *Lachnospiraceae_NK4A136_group, Bacteroides*, and *Alistipes* in the Model group showed independent distribution, forming a closed-loop network that represents “niche monopoly.” The LT2 and control groups had some of the same microbiota, such as *Muribaculum* and *Ruminococcus*. This is shown by the lines connecting the two groups and pointing toward the center of the sectors. *Lactobacillus* (Control group) and *Dubosiella* (LT2 group) exhibited topological symmetry, characterized by connecting lines of consistent thickness. The LT2 intervention restored the microbial structure to match that of the Control group by severing the red links between *Lachnospiraceae* and the Model group, while simultaneously strengthening the yellow connections associated with taxa such as *Dubosiella*. The vulnerability of the closed-loop network in the Model group provides a basis for formulating targeted prevention strategies.

## 4 Discussion

The pathophysiology of polycystic ovarian syndrome (PCOS), the most common endocrine and metabolic illness among women of reproductive age, involves complicated interactions among several systems (Geng et al., 2025). To thoroughly examine the intervention effects of lobetyolin in alleviating PCOS phenotypes and its underlying molecular mechanisms, a DHEA-induced PCOS mouse model was established. From a pathophysiological standpoint, hyperandrogenism and insulin resistance are the principal elements of the distinctive characteristics of PCOS (Mohamed et al., 2023; Wang et al., 2019). Insulin resistance causes hyperinsulinemia, which accelerates the progression of the disease in different ways. High insulin levels directly increase the activity of LH-dependent steroidogenic enzymes and the growth of ovarian stromal cells. This leads to an excess of androgens, such as testosterone (Qi et al., 2025). Insulin resistance also lowers the synthesis of hepatic sex hormone-binding globulin (SHBG), further increasing free androgen levels (Song and Choi, 2022). This metabolic-endocrine disruption results in a notable elevation in the LH/FSH ratio and ovulatory failure, creating a positive feedback loop (Pratama et al., 2024; Xia et al., 2023). LT intervention significantly affected the DHEA-induced PCOS model. In DHEA-induced PCOS mice, LT effectively restored HPG axis homeostasis. This was demonstrated by the normalization of serum gonadotropins (LH, FHS) and testosterone, as well as the restoration of regular estrous cycles and enhanced ovarian morphology (Figure 2). Importantly, the hallmark LH elevation was corrected by LT, and the pathological LH/FSH ratio imbalance was reversed. Simultaneously, LT improved metabolic phenotypes and improved insulin sensitivity, as evidenced by a decrease in fasting insulin levels and a smaller HOMA-IR index (Figure 3).

A regulatory network comprising three essential genes in the steroid hormone synthesis pathway-CYP11A1, CYP17A1, and CYP19A1- is involved in the pathophysiology of PCOS (Heidarzadehpilehrood et al., 2022). The conversion of cholesterol to pregnenolone (PREG) is catalyzed by CYP11A1, which is a rate-limiting enzyme in steroidogenesis. According to genomic research, certain single-nucleotide polymorphisms (SNPs; for example, rs11632698) may significantly increase enzyme stability and, consequently, conversion efficiency (Muccee et al., 2022). 17α-Hydroxylase/17,20-lyase, an enzyme with two roles in steroidogenesis, is encoded by CYP17A1, which can convert progesterone into 17α-hydroxyprogesterone and pregnenolone into 17α-hydroxypregnenolone, and further catalyze these intermediates to dehydroepiandrosterone and androstenedione. Consequently, in patients with PCOS, this can worsen hyperandrogenism by increasing the synthesis of androgen precursors in the ovaries and adrenal glands due to increased CYP17A1 activity (Pusalkar et al., 2009). Aromatase, the primary enzyme responsible for converting androgens to estrogens, is encoded by the CYP19A1 gene. This conversion process may be hampered in patients with PCOS due to decreased aromatase activity in ovarian granulosa cells, which could result in intraovarian androgen buildup, maintaining the PCOS phenotype by aggravating ovulatory failure and hyperandrogenism (Yu et al., 2013). CYP11A1 catalyzes the conversion of cholesterol to pregnenolone in the steroidogenesis pathway, supplying crucial substrates for CYP17A1 (Singh et al., 2022). The dual enzymatic activity of CYP17A1 (17α-hydroxylase/17,20-lyase) is pathologically increased in patients with PCOS, significantly increasing the synthesis of androstenedione and dehydroepiandrosterone (Hu X. et al., 2023). Simultaneously, ovarian granulosa cells exhibit epigenetically decreased CYP19A1 (aromatase) activity, which hinders the conversion of androgens to estrogens (Ke et al., 2025). Systemic and intraovarian hyperandrogenemia, which presents clinically as hirsutism, acne, and ovulatory failure, results from this dual dysregulation. By downregulating CYP11A1 expression, blocking CYP17A1 transcription, and upregulating CYP19A1 expression in mouse ovarian tissue, our study showed that lobetyolin can effectively break this harmful cycle. LT establishes the molecular foundation for its phenotypic amelioration in PCOS by reducing serum testosterone levels as a result of its dual “upstream inhibition and downstream activation” regulatory pattern. The qPCR detection of steroidogenic enzyme genes (CYP11A1, CYP17A1, CYP19A1) was limited by the small sample size (*n* = 3), precluding precise capture of the multi-layered complexity of enzyme activity regulation. It should be particularly noted that a significant disconnect exists between mRNA expression and functional enzyme activity. Consequently, the current transcriptional data with *n* = 3 can only suggest that LT may regulate the transcriptional level of the androgen synthesis network, but cannot confirm its functional consequences.

The “dual-track parallel” feature of chronic inflammation in PCOS is that systemic increases in IL-1β, IL-6, and TNF-α are positively correlated with free testosterone levels (Wang et al., 2024; Liu et al., 2025), whereas localized ovarian infiltration by macrophages and lymphocytes disturbs the immune microenvironment (Gonzalez et al., 2018; Tarkun et al., 2006). These inflammatory mediators decrease CYP19A1 expression, activate the NF-κB/NLRP3 inflammasome, and aid in the activation of insulin receptor Ser307 via the JNK pathway (Liu et al., 2025). This study showed that by blocking the TLR4/NF-κB(p65) pathway, lobetyolin decreased ovarian production of IL-6 and TNF-α and halted the inflammatory cascade.

However, the small sample size limits precision in local cytokine quantification; thus, these findings should be regarded as exploratory and require validation using laser-capture microdissection or single-cell approaches to account for follicular heterogeneity.

Gut dysbiosis is a common feature observed in rodent experimental models and individuals with PCOS. Gut microbiota may actively contribute to and intensify inflammatory processes in patients with PCOS, possibly impairing the function of the intestinal mucosal barrier. This can result in systemic metabolic disruptions, further contributing to the pathophysiology of PCOS (Zeng et al., 2019). According to a study by (Lin et al. 2021), mice administered DHEA along with a high-fat diet showed a considerable increase in *Firmicutes* and a corresponding decrease in *Bacteroidetes* in their fecal microbiota. To disrupt the “dominant taxa monopoly” exhibited in the Model group and restore colonization resistance, this study found that lobetyolin efficiently increased microbial α-diversity indices, increasing species richness and enhancing community evenness. Notably, lobetyolin reversed the pathological cycle marked by a high F/B ratio, which promotes hyperactive energy uptake and ultimately results in metabolic problems by selectively decreasing the abundance of *Firmicutes* while increasing that of *Bacteroidetes*. The lobetyolin-specific biomarker, *Dubosiella*, was discovered using linear discriminant analysis (LDA). It showed topological symmetry with *Lactobacillus* in the Control group, indicating a possible functional compensatory mechanism between both species to preserve microbiota homeostasis.

According to recent studies (Wang et al., 2022), *Dubosiella* abundance was significantly lower in PCOS mouse models. Notably, acylcarnitine metabolites, which are important mediators connecting estrogen insufficiency to lipid metabolism problems, were tightly associated with *Dubosiella* abundance in ovariectomized (OVX) mice models. This finding implies that *Dubosiella* may be essential for controlling metabolic homeostasis, which is dependent on ovarian hormones (Guo et al., 2023). In addition, studies have found that *Dubosiella* plays a role in the occurrence and development of PCOS through multiple pathways, such as affecting intestinal barrier function, short-chain fatty acid production, inflammatory pathways, and bile acid metabolism (Kong et al., 2024; Qian et al., 2026). However, there is currently no direct evidence that a single colonization of *Dubosiella is* sufficient to completely reverse the PCOS phenotype. Future studies should further clarify the causal relationship between *Dubosiella* and the pathogenesis of PCOS and its specific molecular mechanisms through methods such as germ-free mouse reconstitution experiments and integrated analyses of metabolomics and metagenomics. Through β-glucuronidase activity, LT-enriched *Muribaculum* sp. may control enterohepatic circulation, encourage the reabsorption of estrogen metabolites, and indirectly improve local estrogen production and balance ovarian metabolism (Zeibich et al., 2021). By activating GPR43/41 receptors, commensal bacteria, such as *Muribaculum* sp. and *Paramuribaculum intestinale*, whose metabolic byproducts, short-chain fatty acids (SCFAs), suppressed NF-κB-mediated proinflammatory responses and decreased serum IL-6 and TNF-α levels, were enriched following LT administration (Zhu et al., 2024; He et al., 2020; Peng et al., 2024; Luo et al., 2021). The gut microbiota profile of the Model group was distinctive, showing notable enrichment of *Alistipes_sp*. and *Lachnospiraceae_NK4A136_group*. The *LachnospiraceaeNK4A136* group promotes peripheral androgen production by altering the intestinal metabolism of androgen precursors (Liu et al., 2020). Increased abundance of the branched-chain amino acid (BCAA) producer. *Alistipes* sp. is associated with higher levels of circulating BCAAs, which are known risk factors for insulin resistance (Pedersen et al., 2016; Li et al., 2022).

In mouse models of PCOS, abnormal bile acid profiles are closely associated with the pathophysiology of the disease (Wang et al., 2026). In the DHEA-induced PCOS model, recent studies have identified a distinctive “triple aberration” in bile acid metabolism: a marked increase in 3-α-OH-7-O-5β-CA (signaling hyperactivation of the CYP7A1-mediated classical synthesis pathway) (Li et al., 2023), a synchronized decrease in alternative pathway products (ω-MCA, α-MCA, and 3β-MCA, indicating suppressed CYP27A1/CYP7B1 activity) (Xing et al., 2023). Hepatic lipid accumulation is strongly correlated with this metabolic problem, which may worsen PCOS progression by interfering with FXR/TGR5 dual-receptor signaling (Li Q. et al., 2025). Through a “dual-track remodeling” strategy, lobetyolin specifically elevates the levels of glycine-conjugated bile acids (such as glycohyodeoxycholic acid (GHDCA), glycolithocholic acid (GLCA), and glycodeoxycholic acid (GDCA), which fortify intestinal barrier integrity (Ma et al., 2025). Concurrently, LT promotes the generation of isoleithocholic acid (ILCA), which improves insulin sensitivity via the TGR5 pathway (Ye et al., 2026). Interestingly, lobetyolin specifically increases taurochenodeoxycholic acid (TCDCA) and hyocholic acid (HCA) levels. TCDCA activates the FXR-SHP pathway to reduce SREBP-1c, which lowers androgen precursor production (He et al., 2024), whereas HCA acts as a TGR5 agonist and FXR antagonist, working in concert to increase GLP-1 expression (Zheng et al., 2021). These data indicate that LT may exert a preventive effect on PCOS by modulating bile acid metabolism, providing a basis for the potential of LT in preventing PCOS; nonetheless, conclusive causal linkages and specific target interactions necessitate additional mechanistic exploration. Moreover, while our data indicates that LT has preventive effects against PCOS and can alter gut microbiota composition and fecal bile acid profiles, the present experimental design does not allow for conclusive determinations regarding the causative relationships among these three factors. It is still unclear whether changes in microbiota directly cause changes in bile acids, whether bile acids have an effect on the microbiota, or whether both are outcomes of LT therapy. Subsequent investigations utilizing fecal microbiota transplantation, germ-free animal models, or targeted bacterial colonization are necessary to elucidate the exact function of the microbiota-bile acid axis in facilitating LT's protective benefits against PCOS.

Angiogenesis is important in the reproductive process, from folliculogenesis to luteal function. During the follicular phase, VEGF facilitates the formation of a mature peri-follicular vascular network that delivers vital nutrients, oxygen, and paracrine hormonal signals to granulosa and theca cells, ensuring the maintenance of follicular microenvironment homeostasis (Coppola et al., 2005). Vascular growth is important in establishing the luteal phase. A large capillary network delivers nutrients, hormones, and cholesterol to luteal cells (Kurowska et al., 2020). In individuals with PCOS, VEGFA expression in ovarian tissue and follicular fluid is markedly increased, which is strongly correlated with enhanced ovarian stromal vascularization and atypical proliferation of the inner theca layer (Stassi et al., 2019). Additionally, stimulation of the SOCS3/STAT3/VEGFA pathway enhances ovarian angiogenesis (Hu J. et al., 2023). VEGFR2, the main receptor that mediates VEGF angiogenic effects, is significantly expressed in the granulosa, theca, and stromal cells of the ovaries of patients with PCOS. Increased expression of this gene creates a positive feedback loop with VEGFA, worsening ovarian angiogenesis dysregulation (Artini et al., 2006). Furthermore, the expression levels of MMP-2 and MMP-9 in the follicular fluid and granulosa cells of individuals with PCOS are markedly elevated, which is associated with follicular developmental abnormalities and ovulatory dysfunction (Butler et al., 2025). High MMP-2/9 levels are correlated with hyperandrogenism, insulin resistance, and follicular atresia (Butler et al., 2025). In PCOS, increased MMP-9 levels may facilitate unregulated angiogenesis, resulting in cyst development (Jankowska-Ziemak et al., 2025). VEGF specifically causes endothelial cells to release MMP-9, which breaks down the basement membrane and allows cells to move, creating the VEGFA-VEGFR2-MMP2/9 signaling axis, and the excessive activation of this axis leads to ovarian angiogenesis dysregulation, structural disruption of follicular walls, and cyst development, which are significant molecular mechanisms in the pathophysiology of PCOS (Jankowska-Ziemak et al., 2025). This study showed that the levels of VEGFA, VEGFR2, and MMP2/9 were much higher in the ovarian tissues of mice in the Model group than in the ovarian tissues of mice in the experimental group after LT intervention. These findings align with prior research, in patients with PCOS and animal models, ovarian VEGFA is markedly increased in follicular fluid, serum, and ovarian tissues, whereas VEGFR2 is predominantly expressed in theca and granulosa cells, collectively facilitating ovarian stromal vascularization and aberrant growth of the theca interna (Huang and Wang, 2020). Simultaneously, higher levels of MMP2/9 cause excessive breakdown of the extracellular matrix, weakening the follicular wall structure and contributing to cyst formation (Grzesiak et al., 2022; Wang et al., 2025). Mechanistically, LT intervention successfully reconstituted the ovarian angiogenic milieu in PCOS mice by concurrently inhibiting VEGFA-VEGFR2-mediated pathological angiogenesis and MMP2/9-dependent extracellular matrix remodeling. Despite these advancements, the causal link between microenvironmental restoration and the subsequent stabilization of follicular development, ovulatory function, ovarian shape, and endocrine balance requires experimental confirmation.

Patients with PCOS, which is characterized by a “triple pathological network” of increased oxidative stress, an unbalanced inflammatory microenvironment, and aberrant vascular remodeling, have dysregulated alterations in uterine molecular markers, which constitute the primary pathological basis for pregnancy failure (Ding et al., 2025; Gao et al., 2025). The lipid peroxidation product MDA accumulated in the endometrium of DHEA-induced PCOS model mice, which showed a classic phenotype of aberrant alterations in uterine molecular markers and a highly degraded oxidative defense mechanism. This aligns with clinical observations in patients with PCOS, in whom elevated endometrial ROS levels lead to dysregulated expression of uterine molecular markers (Shan et al., 2022). Redox homeostasis supports the formation of pinopodes and the establishment of endometrial epithelial cell polarity during embryo implantation. Following LT intervention, GSH and SOD activities in the uterus of PCOS mice significantly increased, whereas MDA levels significantly decreased. The significantly higher endometrial levels of proinflammatory cytokines (IL-6, TNF-α) in the model group are consistent with a pathogenic cascade that includes dysbiosis of the gut microbiota, LPS translocation, and activation of the TLR4/NF-κB signaling pathway (Li C. et al., 2025). In this study, the PCOS-related abnormally accelerated angiogenesis-stromal remodeling process in the endometrium was effectively reversed by LT. Conversely, the LT-treated group showed a significant increase in TIMP1/TIMP2 levels and a decrease in VEGFA and VEGFR2 expressions. Conversely, the LT-treated group showed a significant increase in TIMP1/TIMP2 levels and a decrease in VEGFA and VEGFR2 expressions. Although our findings indicate enhancements in molecular markers of uterine receptivity, functional reproductive outcomes (such as implantation rates and pregnancy success) were not evaluated in this study and require further investigation. While our findings indicate enhancements in molecular markers of uterine receptivity, functional reproductive outcomes (such as implantation rates and pregnancy success) were not evaluated in this study and require additional examination. Due to the heightened sensitivity of the uterus to sex hormones compared to the ovary, qPCR analysis of uterine remodeling markers was constrained by limited sample size. The small sample (*n* = 3) precluded balanced distribution across estrous cycle stages, rendering cycle confounding a potential source of bias in estimating LT effects that necessitates cycle-stratified validation. Nevertheless, these data hold clear scientific value: LT may alleviate PCOS-associated endometrial hyperplasia and vascular dysfunction by suppressing VEGFA/VEGFR2-mediated angiogenic signaling and MMP2/9-dependent matrix degradation; collectively with ovarian histological improvements and gut microbiota modulation, these findings form a multi-dimensional regulatory mechanism supporting the potential of LT for PCOS prevention.

## 5 Conclusion

In summary, lobetyolin, a chemical derived from *Codonopsis pilosula*, successfully interrupted the detrimental cycle of hyperandrogenism, insulin resistance, and persistent low-grade inflammation in DHEA-induced mice with PCOS. This effect is achieved via combinatorial control of the interconnections among metabolism, inflammation, microbiota, and uterine receptivity. LT inhibits CYP11A1/CYP17A1-mediated androgen synthesis and promotes CYP19A1-dependent aromatization by targeting ovarian steroidogenic enzymes. This corrects hyperandrogenism by reducing testosterone production and promoting its conversion to estradiol. By changing the “*Dubosiella*–*Muribaculum*” symbiotic microbiota and restoring the bile acid profile, TLR4/NF-κB pathway activity is indirectly suppressed, which breaks the microbiota-inflammatory axis. Lobetyolin also fixes the endometrial vascular milieu by bringing the levels of MMPs and TIMPs back into balance. Consequently, LT represents a comprehensive intervention strategy for PCOS prevention, simultaneously targeting hyperandrogenism, insulin resistance, chronic inflammation, and uterine molecular marker abnormalities, demonstrating its potential for clinical application and precision nutritional prevention.

## Data Availability

The datasets presented in this study can be found in online repositories. The names of the repository/repositories and accession number(s) can be found in the article/Supplementary material.
