# Supplementary material for: Lobetyolin reshapes gut microbiota and bile acid metabolism to improve androgen-driven PCOS phenotypes in mice
Source: Front Microbiol. 2026 May 1;17:1810261. doi: 10.3389/fmicb.2026.1810261 (PMC13176234; doi:10.3389/fmicb.2026.1810261)
Supplement: Supplementary file 1 [file Table_1.docx]

Suplementary Table 1 Primers for qRT-PCR

| Gene Forward Primer Reverse Primer Product  Length(bp) |
| --- |
| VEGFA CTGCTGTCTTGGGTGCATTG CACCGCCTTGGCTTGTCA 132  VEGFAR2 GGAGCAGAAAGACGGTGATG TCTGGTGTTGGCACAGAACT 127  MMP2 CTTGATGACGGCCTTGAAGA CAGCCGTAGAAGGTGGTGAG 118  MMP9 CTTCCAGTACCGAGAGAAAGC GCTGCTTCTCTCCCATCATC 125  TIMP1 ACAGCTATGGGTTCCGTGTG CCAGTTTGATGTTGCAGGGG 121  TIMP2 GCTGGACGTTGGAGGAAAGA CAGGCAGTGATGTAGACAGGAA 114  CYP11A1 GGCACAGAGTACCTGGTGAA GTGTCCAGGATGAGGAAGCA 130  CYP17A1 CCTGCTACTTGCTGCTCTGG GGGTGAAGGTGTTGAGCAGA 123  CYP19A1 TCTGACGCAGAGTGCAGAGA GGACATGGAAACGGTGGTAG 135  IL-6 CTGCAAGAGACTTCCATCCAG AGGTGCCGTCTGTGGTGTTA 118  TNF-a TATGGCCCAGACCCTCACAC GGGCTTCTCACACACGATCA 122  TLR4 CTGGCATGGCTTACACCAGT CAGGGCTTTTCTGAGCGTTA 120  NF-κB(p65) CGGCTATGAGCTCAAGATGC TGGGTGCGTCTTAGTGGTAT 128  β-actin GTGACGTTGACATCCGTAAAGA GTAACAGTCCGCCTAGAAGCAC 141 |
