# Supplementary material for: Lobetyolin reshapes gut microbiota and bile acid metabolism to improve androgen-driven PCOS phenotypes in mice
Source: Front Microbiol. 2026 May 1;17:1810261. doi: 10.3389/fmicb.2026.1810261 (PMC13176234; doi:10.3389/fmicb.2026.1810261)
Supplement: Supplementary file 2 [file Table_2.docx]

Supplementary Table 2 Comparison of glycine-conjugated bile acid levels

|  | Model | LT2 | *P* |
| --- | --- | --- | --- |
| GHDCA  GCDCA  GDCA  GLCA  GCA  GUDCA | 104.70±9.79  12.88±4.93  420.20±28.38^b^  10.10±1.12^B^  4898±4261  57.92±8.85^ab^ | 226.40±44.56  23.72±4.53  989.20±235.10^a^  43.90±8.88^A^  5238±1948  76.35±17.28^a^ | 0.02  0.19  0.03  0.0014  0.99  0.53 |
